# Sweet’s syndrome with Koebner phenomenon triggered by G-CSF as a preleukemic manifestation in a patient with primary myelofibrosis: a case report

**DOI:** 10.3389/fmed.2025.1632767

**Published:** 2025-11-10

**Authors:** Zhiyuan Zhang, Dan Zhou, Yahui Liu, Lingyu Meng, Bai Ji

**Affiliations:** 1 Department of Colorectal and Anal Surgery, General Surgery Center, The First Hospital of Jilin University, Changchun, China; 2 Department of Hepatobiliary and Pancreatic Surgery, General Surgery Center, The First Hospital of Jilin University, Changchun, China

**Keywords:** Sweet’s syndrome, G-CSF, Koebner phenomenon, leukemia, VEXAS, primary myelofibrosis

## Abstract

**Background:**

Sweet’s syndrome (SS), also known as acute febrile neutrophilic skin disease, is a rare inflammatory skin disease. Clinically, patients often have fever and leukocytosis, characterized by painful erythema, papules or pustules. According to different etiologies, SS can be classified into three types: idiopathic, tumor-related and drug-induced. Among them, tumor-associated SS is closely related to some hematological malignancies. Drug-induced SS, on the other hand, can be induced by the use of granulocyte colony-stimulating factor (G-CSF).

**Case introduction:**

This article reports a 42-year-old male patient who was admitted to the hospital due to “significant splenomegaly and splenic stasis.” After admission, the patient was diagnosed with primary myelofibrosis through examination. During the hospitalization process, the patient received G-CSF treatment and open total splenectomy. On the 8th day after subcutaneous injection of 100 μg of G-CSF, erythema, swelling and superficial ulcers occurred at the surgical incision and drainage site, accompanied by Koebner’s phenomenon. The results of skin biopsy indicated Sweet’s syndrome. The rash improved significantly and rapidly several days after intravenous application of methylprednisolone. However, several months after discharge, the patient was diagnosed with acute myeloid leukemia through bone marrow puncture in another hospital and unfortunately passed away due to the progression of the disease.

**Conclusion:**

Among patients with complex underlying diseases such as myelofibrosis and receiving G-CSF treatment, Sweet’s syndrome may not only be an adverse reaction caused by drugs, but more likely to be an early manifestation of leukemia. At this time, SS is a complex comprehensive disease caused by multiple factors. It is not advisable to simply diagnose its phenotype. Instead, a comprehensive assessment should be conducted to reduce misdiagnosis and missed diagnosis. Therefore, in clinical practice, we should strengthen multidisciplinary collaboration even more and enhance the understanding of Sweet’s syndrome itself and its role as a potential early warning signal of leukemia. This enables the early identification of diseases and the adoption of effective treatment measures. Although the Koebner phenomenon is relatively rare in SS, its occurrence should be highly valued.

## Introduction

Sweet’s syndrome (SS), first described by Robert Douglas Sweet in 1964 (2), is an acute inflammatory disease characterized by skin damage, mainly manifested as painful erythema, papules, pustules or nodules, usually accompanied by fever and neutropenia. Its main pathological feature is the extensive infiltration of neutrophils in the dermis (1, 3). Although the etiology of SS is not yet fully understood, current studies have shown that some factors such as drugs, tumors and infections may induce this disease (1, 4). Drugs are one of the important causes. In particular, granulocyte colony-stimulating factor (G-CSF) has been reported many times as one of the common triggers of SS (5, 6). This case report describes a 42-year-old male patient who was admitted to the hospital due to “significant splenomegaly and splenic congestion.” The patient suffered from primary myelofibrosis and a significant reduction in the three lineages of blood cells. Shortly after the treatment with G-CSF, typical Sweet syndrome manifestations appeared on the skin. The skin histopathology results showed a diffuse infiltration of a large number of neutrophils in the dermis, which was considered as SS. After glucocorticoid treatment, the patient’s skin lesions improved significantly and gradually healed. However, in the subsequent follow-up, we learned that the patient was diagnosed with acute myeloid leukemia (AML) 8 months after the onset of SS and eventually died due to the continuous deterioration of the condition. The SS of this patient was initially considered to be induced by the use of G-CSF. However, the patient suffered from primary myelofibrosis and was diagnosed with acute myeloid leukemia 8 months after the onset of SS. Therefore, we believe that the SS of this patient is more likely to be an early manifestation of a potential malignant hematological disease. This case emphasizes that for individuals with underlying diseases such as bone marrow lesions or abnormal immune function, when Sweet’s syndrome occurs after the use of G-CSF, in addition to considering the factor of drug induction, more attention should be paid to its potential as an early warning signal of hematological malignancies.

## Case introduction

**FIGURE 1:**
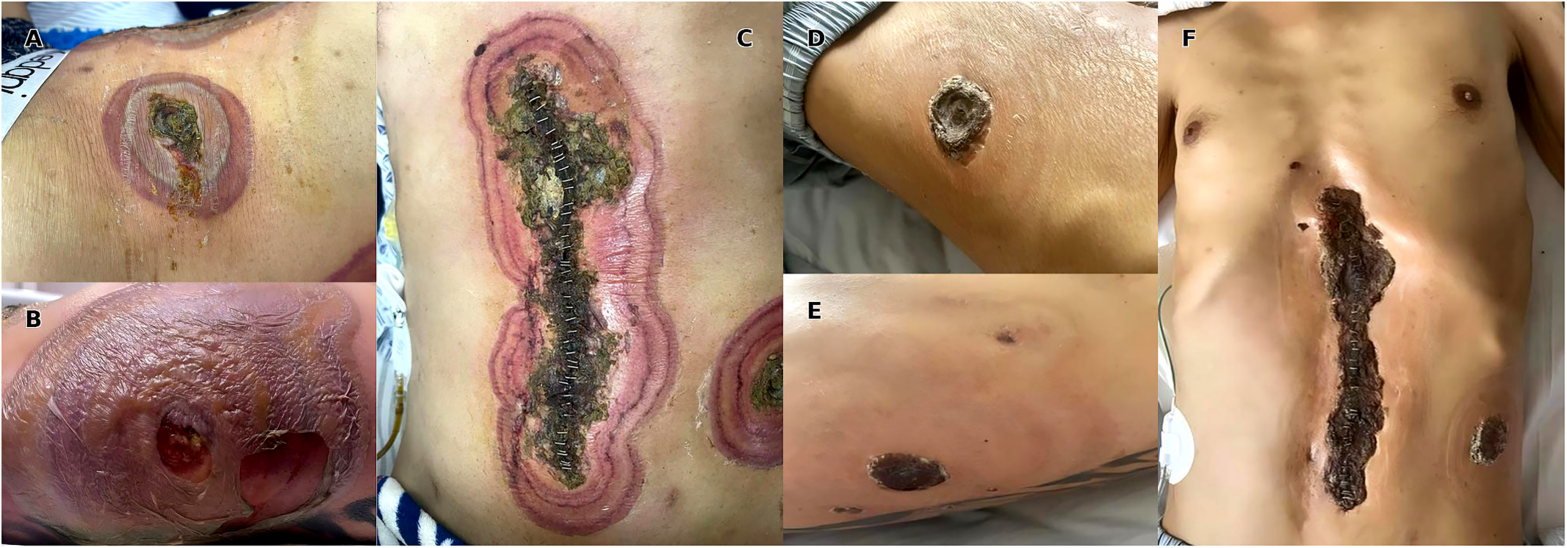
Postoperative erythema, swelling, and ulceration around the surgical incision and drainage tube sites, demonstrating the Koebner phenomenon. **(A)** Erythema and ulceration radiating from the drainage tube site on the patient’s left lower abdomen. **(B)** Erythema and ulceration radiating from the drainage tube site on the posterior left flank. **(C)** Erythema and ulceration around the central abdominal incision. **(D)** The skin at the left lower abdominal drainage site is healing well after corticosteroid treatment. **(E)** The skin at the left posterior lateral waist drainage site has a central scab, with surrounding inflammation subsiding. **(F)** The abdominal midline incision shows a scab, with significant improvement in the condition.

**FIGURE 2:**
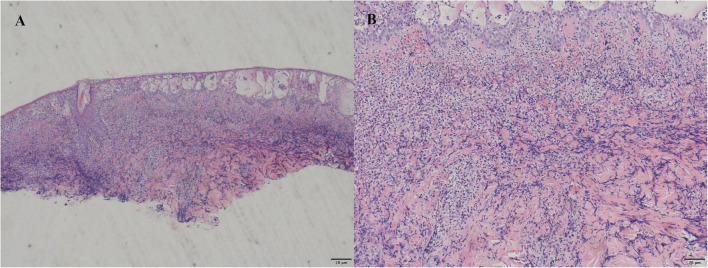
**(A)** The skin biopsy specimen at 40 × magnification shows dermal neutrophilic infiltration and vascular dilatation. **(B)** High-power view (×100) of the same lesion reveals dense neutrophilic infiltration predominantly within the superficial and mid-dermis.

The patient was a 42-year-old male who visited the Department of Hepatobiliary and Pancreatic Surgery of our hospital due to “significant splenomegaly and splenic stasis.” Physical examination showed that the patient was emaciated and the spleen could be palpated under the left costal margin, reaching 5 cm. The initial laboratory examination upon admission indicated that the white blood cell count (WBC) was 3.23 × 10^9^/L, hemoglobin (Hb) was 96 g/L, and platelets (PLT) were 15 × 10^9^/L. Abdominal ultrasound showed that the splenic thickness was 59 mm and the splenic length was 182 mm, suggesting splenomegaly. After admission, symptomatic treatments such as nutritional support and platelet transfusion were given. From the second to the third day of hospitalization, the patient’s white blood cell count continued to decline, with the lowest dropping to 1.56 × 10^9^/L. On the 4th day, subcutaneous injection of G-CSF (100 μg) was given to improve the state of granulocytopenia. Open splenectomy was performed on the same day to control hypersplenism and related compression symptoms. Ertapenem (1 g/day) was given after the operation to prevent infection. However, there was no significant improvement in the peripheral blood images of the patients after the operation, and the reduction of the three lines of cells persisted. To further clarify the cause of cytopenia and rule out bone marrow hematopoietic dysfunction, we sought a consultation in the hematology department and underwent bone marrow puncture examination. The results indicated myelofibrosis. Granulopoiesis accounted for 94.5% of nucleated cells, composed entirely of mature granulocytes exhibiting toxic granulation and cytoplasmic vacuolization. Although vacuoles were not observed in myeloid or erythroid precursors, these findings, in light of the patient’s clinical context, raise suspicion for VEXAS syndrome and merit further evaluation. Molecular biological tests showed that CALR mutation, JAK2-V617F, McL-w515l/K and BCR-ABL were all negative, supporting the diagnosis of primary myelofibrosis. On the 8th day after the operation, the patient presented with elevated body temperature (38.1 °C), and at the same time, erythema, swelling and superficial ulcers appeared on the skin of the left lower abdominal drainage opening, the left lumbar drainage opening and the incision area of the midline of the abdomen, presenting a typical Cobner phenomenon (Figures 1A–C). The reexamination of the blood routine showed that the WBC rose to 14.14 × 10^9^/L, and the proportion of neutrophils was 94%. Skin biopsy was performed after consultation with the dermatology department. Histopathology showed mild irregular hyperplasia, edema and spongy blistering in the epidermis, and a large number of diffuse infiltration of neutrophils could be seen in the dermis (Figure 2).

Combined with the clinical manifestations, the diagnosis was Sweet syndrome (acute febrile neutrophilic skin disease). After diagnosis, intravenous methylprednisolone treatment was given (40 mg in the morning and 20 mg in the evening). One week after the treatment, the color of the rash gradually deepened and the exudation significantly decreased. On the 26th day after the operation, the patient’s condition was stable and he was discharged from the hospital.

At the 2-week follow-up after discharge, the erythema and superficial ulcers at the original incision and drainage port had significantly improved (Figures 1D–F), and no new rash occurred. Methylprednisolone was gradually reduced in dosage and successfully discontinued as the rash improved. During this period, there was no recurrence of symptoms or adverse reactions related to glucocorticoids, and G-CSF was no longer used. The patient gained weight and recovered well overall. He reported no obvious discomfort and expressed satisfaction with the treatment effect.

Eight months after discharge, the patient visited the local hospital again due to symptoms such as persistent fever and fatigue. The blood routine examination indicates anemia and a significant reduction in platelets. Bone marrow puncture examination was performed to determine the cause of the disease. The results showed that the proportion of primitive granulocytes reached 37%, and the granulocyte-erythroid ratio increased (1.92:1), suggesting abnormal proliferation of the granulocyte-lineage. Combined with the clinical manifestations and the morphological characteristics of the bone marrow, the diagnosis was AML. Within the second month after diagnosis, the patient successively developed complications such as infection, electrolyte imbalance and hypoalbuminemia. Despite active anti-infection and supportive treatment, the condition deteriorated rapidly and the patient eventually died due to multiple organ failure.

## Discussion

Sweet’s syndrome also known as acute febrile neutrophilic skin disease, was first proposed in 1964 (2). The typical clinical manifestations of SS include tender erythematous papules, plaques, pustules or nodules, often accompanied by fever and neutropenia. Its main pathological feature is the dense infiltration of neutrophils in the dermis, forming an acute inflammatory response (3). According to different etiologies, SS is classified into three types: idiopathic, tumor-related and drug-induced (1).

Drug-induced SS usually emerges rapidly after the use of specific drugs such as G-CSF and all-trans retinoic acid. The rash is relatively localized. The recovery is good and the recurrence rate is low after drug withdrawal and glucocorticoid treatment (1). In this case, the patient presented with typical skin lesions on the 8th day of treatment with G-CSF, manifested as erythema, blisters, superficial ulcers, accompanied by fever and elevated white blood cells. Skin biopsy confirmed dermal neutrophil infiltration. And the skin lesions improved rapidly after receiving glucocorticoid treatment. It met the five diagnostic criteria for drug-induced Sweet syndrome proposed by Walker and Cohen (5), supporting G-CSF as the main inducing factor of SS in this case. See Table 1 for details.

**TABLE 1:** Diagnostic criteria for drug-induced Sweet’s syndrome (DI-SS).

No.	Diagnostic criteria
1	Sudden onset of painful erythema or nodules.
2	Histopathological evidence of dense neutrophilic infiltration without evidence of leukocytoclastic vasculitis.
3	Fever > 38 °C.
4	Temporal association between drug intake and clinical manifestation or recurrence after drug withdrawal.
5	Improvement of lesions following discontinuation of the drug or systemic corticosteroid treatment.

Colony-stimulating factor is a key hematopoietic regulatory factor and is widely used in the treatment of bone marrow suppression after chemotherapy (7). It can significantly promote the proliferation, differentiation and activation of neutrophils, while enhancing the chemotaxis and tissue infiltration ability of neutrophils (4). Some literature indicates that G-CSF can up-regulate the expression of pro-inflammatory factors such as IL-8, MCP-1, and TNF-α. These pro-inflammatory factors drive neutrophils to migrate to and accumulate in the skin, inducing the typical pathological reactions of SS (7). G-CSF can also activate signaling pathways such as STAT3 and NF-κB to enhance the innate immune response. However, in patients with immune dysfunction or myelofibrosis, the use of G-CSF may further disrupt immune homeostasis, leading to the occurrence of SS (4).

Granulocyte colony-stimulating factor has been widely reported as one of the common triggers of Sweet’s syndrome. Martin et al. confirmed a significant correlation between G-CSF and SS through the analysis of the pharmacovigilance database (8). Kaya et al. reported a case of SS in a child who received autologous stem cell transplantation after the application of G-CSF (6), and Llamas-Velasco et al. also reported the occurrence of SS related to pegfilgrastim (9). A retrospective study on 50 cases of G-CSF-related SS further provided clinical and pathological feature support (7). The median onset time of SS was 7 days. The main manifestations were papules/plaques (74.0%), nodules (32.0%), and blisters/bullae (24.0%). In skin biopsy, diffuse neutrophil infiltration in the dermis (97.8%), leukocytosis (19.1%), and dermal papillary edema (27.7%) were commonly seen. After discontinuing G-CSF and receiving systemic glucocorticoid treatment, most patients achieved significant clinical improvement within 7 days (7).

Although the clinical manifestations and temporal relationship of this patient met the diagnostic criteria of drug-induced Sweet’s syndrome, considering the underlying hematological background of primary myelofibrosis in the past and the subsequent progression of the disease to acute myeloid leukemia. We believe that this patient with Sweet’s syndrome may have more than just a simple adverse drug reaction. It also conforms to the characteristics of malignancy-associated Sweet syndrome (MASS) (10).

Malignancy-associated Sweet syndrome (MASS) is an important subtype of Sweet syndrome, accounting for approximately 15%–20% of all SS cases (10). It is most commonly found in patients with hematological malignancies, especially AML and myelodysplastic syndrome (MDS) (11, 12), and can also be seen in chronic myeloid leukemia (CML), chronic myelomonocytic leukemia (CMML), and some solid tumors (12, 13). One of the major clinical features of MASS is that skin manifestations often precedes tumor diagnosis (11–13). Therefore, in some cases, it can be regarded as an early signal of potential tumors. At present, there is no unified international diagnostic standard for MASS. Clinically, it is prone to be misjudged as drug allergy or infectious rash, especially in patients after using hematopoietic stimulating factors such as G-CSF (10, 14).

The pathogenesis of MASS has not been fully clarified. Relevant reports suggest that it may be related to the abnormally elevated levels of pro-inflammatory factors such as IL-1, IL-6, TNF-α, and G-CSF. Its pathogenesis is also closely related to neutrophil dysfunction and tumor-related immune disorders (12, 14). In hematological malignancies, the bone marrow microenvironment remains in a state of chronic inflammation for a long time. The continuous release of inflammatory factors can trigger abnormal activation of neutrophils and tissue infiltration, thereby inducing SS (12).

Multiple literatures have revealed the close association between MASS and hematological malignancies, and some studies have proposed that it may serve as an early cutaneous manifestation of certain hematological malignancies. A study by Zhang et al. showed that among 37 SS patients, 27% had hematological malignancies, including AML, MDS, and multiple myeloma (15); Markova et al. analyzed and pointed out that Sweet’s syndrome can be one of the initial symptoms of AML (16); Furthermore, Cohen et al. found that among 108 patients with Sweet’s syndrome, approximately one-quarter developed SS prior to a cancer diagnosis, particularly those associated with AML and MDS, with 16 and 23 cases, respectively (13).

The patient we reported had a basic hematological history of primary mymyfibrosis (PMF), presented with typical SS skin manifestations after receiving G-CSF treatment, and was diagnosed with AML 8 months later. This progression process highly suggests that SS may not be merely an adverse drug reaction induced by G-CSF, but rather should be regarded as a clinical manifestation of MASS. PMF itself, as a myeloproliferative tumor, keeps the hematopoietic system of patients in an abnormally activated state for a long time and may induce SS lesions under the action of inflammatory factors. The underlying hematopoietic lesions may make patients more sensitive to exogenous stimuli such as G-CSF, further promoting the activation of neutrophils and tissue infiltration. To further clarify the classification type of Sweet syndrome in this case, we conducted a comparative analysis of the clinical characteristics of drug-induced SS (DI-SS) and malignancy associated SS (MASS) in previous literature. There are certain differences between the two in terms of inducing factors, onset sequence, systemic manifestations, laboratory tests, skin pathological features, treatment responses and prognosis. Based on the background of the onset, clinical evolution and subsequent development into acute myeloid leukemia of this case,it is more inclined to be tumor-associated SS. See Table 2 for details.

**TABLE 2:** Key differences between drug-induced Sweet’s syndrome (DI-SS) and malignancy-associated Sweet’s syndrome (MASS).

Diagnostic feature	DI-SS	MASS
Common triggers	G-CSF, antibiotics, antiepileptic drugs (5, 7)	AML, MDS, multiple myeloma, solid tumors (10, 13, 15, 16)
Onset timing	Within 1–14 days after drug administration (5, 7)	Before, during, or after malignancy diagnosis (10, 13, 15)
Clinical manifestations	Localized lesions, mild systemic symptoms, possible fever (5, 7)	Often accompanied by fever, hepatosplenomegaly, anemia, fatigue; more severe course (13, 15, 16)
Laboratory findings	Neutrophilia, normal platelets, mild anemia (7)	Pancytopenia or increased blasts, possible leukemic cells (13, 15, 16)
Histopathology	Dense dermal neutrophilic infiltration, no atypia (7)	Similar findings, may show atypical cells or tissue necrosis (15, 16)
Treatment response	Drug withdrawal plus corticosteroids; improvement within 7 days (7)	Often requires treatment of underlying malignancy; corticosteroid response variable; frequent relapse (7, 13, 15, 16)
Prognosis and Follow-up	Favorable prognosis, low recurrence (7)	Indicates tumor activity or progression; poorer prognosis (10, 15, 16)
Other features	Strong temporal link to drug use, no cancer history (5, 7)	Often associated with known malignancy or hematologic abnormalities; prolonged and refractory course (7, 13, 15, 16)

Another notable feature of this case is the occurrence of the Koebner phenomenon, which is manifested as the appearance of new skin lesions consistent with the primary lesion at the skin damage sites such as surgical incisions and drainage ports. This phenomenon suggests that the patient’s skin is more reactive to external stimuli. Although Koebner’s phenomenon is relatively uncommon in Sweet’s syndrome, several reports have documented its occurrence at various sites of cutaneous injury or inflammation, including minor trauma, surgical incisions, cat scratches, biopsy sites, contact dermatitis, insect bites, venous catheter insertions, radiotherapy fields, and venipuncture sites (17, 18). Kluger et al. reported a case of SS induced after tattooing, suggesting the role of skin trauma in the pathogenesis of SS (2); Hiraiwa et al. also described the occurrence of SS lesions at the site of cat scratches, further supporting the existence of the Koebner phenomenon in SS (18). The Koebner phenomenon is relatively common in psoriasis, lichen planus and vitiligo, with different mechanisms: In psoriasis, keratinocytes release antimicrobial peptides to activate TLR9, thereby triggering an immune response along the IL-23/IL-17 axis (19); Lichen planus involves MHC-I expression and CD8+ T cell-mediated epidermal damage (20). In SS, the Koebner phenomenon may be caused by the release of inflammatory factors due to local stimulation, resulting in focal infiltration of neutrophils and thus forming typical lesions (4). This mechanism suggests that the Koebner phenomenon is not limited to T-cell-mediated diseases but can also be seen in neutrophil-related skin lesions.

Furthermore, this case may also be linked to the newly recognized autoinflammatory disorder VEXAS (vacuoles, E1 enzyme, X-linked, autoinflammatory, somatic) syndrome (21). This adult-onset syndrome, caused by somatic mutations in the UBA1 gene, primarily affects males and is characterized by systemic inflammation, hematologic abnormalities, and distinctive bone marrow vacuolization (22). In this case, although vacuolization was observed in mature neutrophils rather than precursor cells and UBA1 genetic testing could not be performed due to the patient’s death, the clinical presentation—including systemic inflammation, hematologic abnormalities, and disease progression to AML—remains highly suggestive of VEXAS syndrome. This case contributes to a more comprehensive understanding of VEXAS syndrome among clinicians, particularly in adult males presenting with systemic inflammatory manifestations and hematological abnormalities. Given the potential clinical overlap between Sweet’s syndrome and VEXAS, establishing an accurate differential diagnosis is of critical importance. Enhancing recognition of both disorders in terms of their clinical presentation, pathological features, and disease progression can help prevent misdiagnosis or missed diagnoses.

This case suggests that SS in a specific clinical context may not only be a manifestation of adverse drug reactions, but also be an early clinical signal of potential hematological malignancies, especially leukemia. For patients with autoimmune diseases, chronic inflammatory states or those who have undergone major surgical operations, risk assessment should be conducted before the application of G-CSF, and close monitoring should be carried out during the treatment process. Once SS is diagnosed, glucocorticoid intervention should be given in a timely manner, and at the same time, vigilance should be exercised against possible underlying hematological diseases. The progression process of this case emphasizes that the diagnosis and treatment of SS should not be limited to the skin manifestations themselves. Instead, it should involve multidisciplinary collaboration such as hematology to enhance the ability to identify and intervene in potential malignant diseases. The patient’s eventual death due to the progression of acute myeloid leukemia further highlights the importance of conducting systematic follow-up and etiological tracking for SS patients.

## Data Availability

The original contributions presented in this study are included in this article/supplementary material, further inquiries can be directed to the corresponding author.
